# The effect of *Moringa oleifera* crude extract on liver cell line, HepG2

**DOI:** 10.1186/s12906-023-04181-8

**Published:** 2023-10-26

**Authors:** Boluwatife O. Sowunmi, Martin Gonzo

**Affiliations:** University of Greenwich, London, UK

**Keywords:** Liver diseases, Medicinal plants, *Moringa oleifera*, Hepatoprotective, Seed extracts, Cell culture, Cell viability

## Abstract

**Background:**

The liver plays a crucial role in the body's metabolic and detoxification processes. Given its importance, compromised liver function can negatively impact the body’s metabolic and physiological function. Liver diseases can result from several factors, including exposure to toxins, alcohol consumption, and viral infections. Therefore, finding natural remedies for liver protection and treatment is important. *Moringa oleifera* is a tree known for its various medicinal properties, including hepatoprotective effects. This study aimed to investigate the potential of *M. oleifera* seed extract in protecting liver cells.

**Methods:**

In this study, dried-seed powder of *M. oleifera* was extracted using extraction solvents, methanol, and ethanol. HepG2 cells were cultured and treated with different concentrations of the extracts. The antioxidative activity, cell viability, and antiproliferation were assessed using the total antioxidant capacity assay (TAC) and 3-(4,5-dimethylthiazol-2-yl)-2,5-diphenyl-2H-tetrazolium bromide (MTT) assay. Additionally, liver enzyme activity was determined through alkaline phosphatase and aspartate aminotransferase activity assays.

**Results:**

The extracts had varying effects on liver cells depending on the concentration and time of exposure. Lower concentrations (50 mg/l and 100 mg/l) have mild stimulatory effects/minimal impact on metabolic activity, while higher concentrations (200 mg/l and 400 mg/l) tend to decrease metabolic activity, especially at later time points. Moreover, the extracts effectively reduced the levels of the liver enzyme AST, indicating their ability to mitigate liver injury.

**Conclusion:**

The study concludes that the crude extracts of *M. oleifera* seeds exhibit potential as a natural remedy for liver diseases. The effects of *M. oleifera* extract suggest that it has potential as a preventive and therapeutic agent for liver damage. This study highlights the importance of exploring natural remedies for liver protection and treatment.

## Background

The liver plays a crucial role in the human body; it is responsible for synthesizing glucose, plasma proteins, clotting factors, and urea; storing glycogen, fat, and vitamins; regulating amino acids; and producing bile for metabolizing fat [1–4]. Liver diseases encompass a range of serious conditions, such as acute or chronic hepatitis, hepatosis, and cirrhosis. These diseases can be caused by several factors, with drug toxicity being a significant contributor. Other common causes of liver diseases include viral infections (such as hepatitis B and C), excessive alcohol consumption, autoimmune disorders, metabolic disorders, and genetic factors [5–7]. Complications such as hepatic encephalopathy, ascites, and variceal bleeding can arise from liver diseases, leading to liver failure if left untreated [8, 9].

Medicinal plants have been explored for centuries as a natural remedy for liver diseases, with various extracts from medicinal plants showing high hepatoprotective activity in animal models. Active components in medicinal plants, such as phenolic, and flavonoid compounds, exhibit antioxidant and anti-inflammatory activities that help obstruct lipid peroxidation and decrease the oxidative stress marker malondialdehyde (MDA) and hepatic marker enzymes, such as aspartate aminotransferase (AST), alanine aminotransferase (ALT), and alkaline phosphatase (ALP) enzymes [10, 11].

*Moringa oleifera* is a vegetable that belongs to the order Brassica and the family Moringaceae and is rich in essential nutrients, amino acids, beta-carotene, antioxidants, vitamins, omega 3 and 6 fatty acids, and minerals [12–16]. Previous studies have investigated the hepatoprotective role of *M. oleifera*, with most focusing on leaf extracts [17–20]. However, there are limited data available on the hepatoprotective role of *M. oleifera* seed extracts. These seeds contain high nutritional content and bioactive compounds. This study investigated the potential of the crude extract of *M. oleifera* seeds to protect liver cells.

## Materials and methods

### Materials

Minimum Eagle’s medium, HepG2 cells, fetal bovine serum (FBS), nonessential amino acids (NEAAs), phosphate-buffered saline (PBS) dimethyl sulfoxide (DMSO) and trypsin solution with 0.25% ethylenediaminetetraacetic acid (EDTA) were obtained from Sigma‒Aldrich (Darmstadt, Germany). The phenolic compound assay, antioxidant assay kit, aspartate aminotransferase (AST) activity assay kit, alkaline phosphatase (ALP) assay kit, and HepG2 cells were obtained from Sigma‒Aldrich (Darmstadt, Germany).

### Seed preparation and extraction

*M. oleifera* seeds were obtained from the natural forest in the northern regions of Namibia. The plant was authenticated by the macroscopic appearance of the seeds, organoleptic testing was also conducted by Professor Habauka M. Kwaambwa of The Namibian University of Science and Technology. The seed samples were collected and washed with distilled water. After the seeds had been cleaned properly, they were air-dried. The seeds were dehusked and 10 g was weighed and ground into powder. The seed powder (10 g) was transferred into a conical flask in preparation for extraction. Each 1 g of powder was macerated with 10 ml of solvent: 80% methanol and 70% ethanol, for 72 h at room temperature. The resulting extract was filtered with Whatman filter paper and vacuum evaporated in a rotary evaporator at 40 °C to obtain a crude extract. The extract was freeze-dried and stored at -20 °C for future use.

### Cell viability assay (MTT assay)

HepG2 cells were thawed and cultured using Dulbecco’s modified Eagle’s medium (DMEM) supplemented with 10% FBS, 1% (v/v) glutamine (200 mM), 1% (v/v) NEAA and 1% penicillin‒streptomycin at 37 °C under an atmosphere of 5% carbon dioxide for 24 h. The cells were washed with phosphate-buffered saline (PBS) and trypsinized using 0.25% trypsin–EDTA solution. A fresh culture medium was added to stop the reaction.

The MTT assay was conducted according to [21] with modifications. Briefly, cells were seeded at 2.5 × 10^5^ cells/ml in 96-well plates for 24 h. The cells were washed with phosphate-buffered saline (PBS) before treatment with *M. oleifera* extracts at different concentrations (50 mg/l, 100 mg/l, 200 mg/l, and 400 mg/l) [21–24]. After 24, 48 and 72 h, the supernatants were collected and transferred into Eppendorf tubes for use in further assays. Fresh culture medium, 100 µl, was added to the wells. MTT reagent, 10 µl, (5 mg/l), was added to the wells and the plates were incubated at 37 °C for 4 h. The resulting formazan salt was dissolved in 50 µl DMSO. The absorbance was measured at 540 nm using a microplate reader. The percentage of the cell viability was determined relative to the control.

### Total phenolic content

Total phenol in *M. oleifera* seeds was measured using a phenolic compound assay kit (Sigma-Aldrich). The assay was conducted according to the manufacturer’s instructions. Briefly, 50 µl of the sample was added to each well and the volume was made up to 100 µl with distilled water (dH_2_O). The PC probe (20 µl) was added to each sample reaction well, except for the background wells. The PC assay buffer (20 µl) was added to the sample background wells. The plate was shaken gently to allow even distribution of the probe. The PC assay buffer (80 µl) was added to all reaction wells and the plate was shaken gently. The plate was incubated at room temperature for 10 min, and the absorbance was read at 480 nm. A catechin standard curve was obtained at different concentrations (0–10 nmol/well, *y* = 0.1671x—0.019, R^2^ = 0.9835).

### Antioxidant capacity assay

Antioxidant capacity was determined using an antioxidant assay kit (Sigma‒Aldrich), and the assay was conducted according to previous studies [25, 26]. In brief, 20 µl of the cell culture supernatant from each treatment was transferred to a separate well of a 96-well plate. The reaction assay mix was added to the assay wells, and the plate was tapped gently to mix and allow even distribution. The plate was then incubated at room temperature for 10 min and the absorbance was measured at 570 nm. A Trolox standard curve was obtained at different concentrations (0–1000 µM, y = 0.0009x + 0.0211, R^2^ = 0.9961).

### Alkaline phosphatase assay

ALP levels were detected using an alkaline phosphatase assay kit (Sigma‒Aldrich). The assay was conducted according to the manufacturer’s instructions. Briefly, 20 µl of cell culture supernatant was transferred into 96well plates. ALP working reagent, 180 µl, was added to the sample wells and the plate was tapped gently to mix. Absorbance was read at 405 nm.

### Aspartate aminotransferase assay

AST enzyme levels were measured using an AST assay kit (Sigma‒Aldrich). Cell culture supernatant (20 µl) obtained from treatment was added to a separate well of 96-well culture plates. The samples were brought to a final volume of 50 µl with AST assay buffer. The reaction mix was added to each well at 100 µl/well. The plate was incubated at 37 °C and the initial absorbance reading was taken after 3 min at 450 nm. The plate was continually incubated, and readings were taken every 5 min until the value of the most active sample was greater than or near the values obtained from the highest standard concentration [27, 28]. A glutamate standard curve was obtained at different concentrations (0–10 nmol/well, y = 0.1164x + 0.0096, *R*^2^ = 0.9966).

### Statistical analysis of experimental data

Microsoft Excel (Version 2307 Build 16.0.16626.20170) 64-bit was used to carry out all the statistical analyses, and the experimental data were analysed. Two-way analysis of variance (ANOVA) was then used to test the mean differences observed among the different times and levels of concentration of the extract.

## Results

### Cell viability and proliferation

**Fig. 1:**
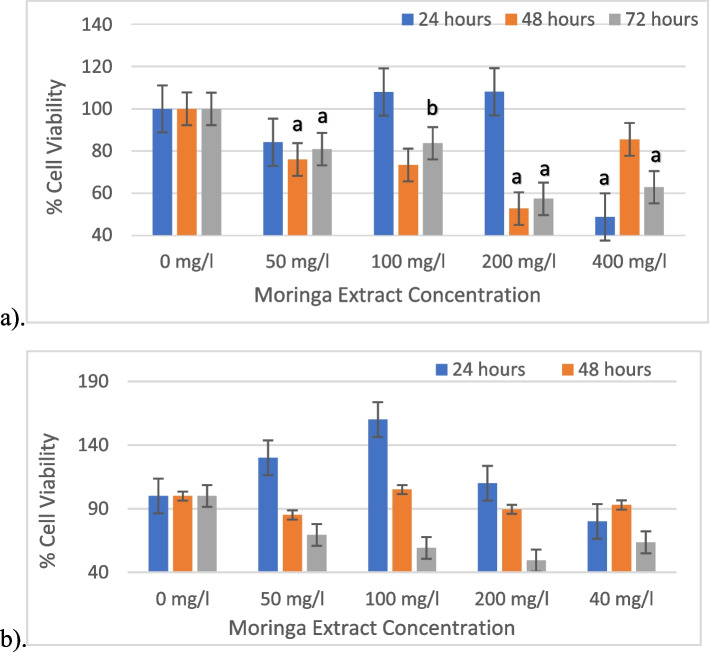
The cell viability results obtained from treatment with **a**). methanolic extract **b**). ethanolic extract at different concentrations for 72 h. Values are the mean ± SD; *a (*P* < 0.05 vs 0 mg/l (control); **b** *P* < 0.01 vs 0 mg/l (control))

The results from the treatment with *M. oleifera* methanolic extract are illustrated in Fig. 1a below. These results indicated that at 24 h, the 400 mg/l concentration significantly reduced cell viability, while the other concentrations (200 mg/l, 100 mg/l, and 50 mg/l) had comparable or slightly higher viability compared to the control. At 48 h, the 200 mg/l concentration exhibited the lowest cell viability, whereas the 100 mg/l and 50 mg/l concentrations showed intermediate viability. By 72 h, the 400 mg/l concentration had the lowest cell viability. The 100 mg/l concentration demonstrated the highest viability compared to the control. The extract exhibited dose-dependent and time-dependent effects on cell viability.

For the ethanol extract, at 24 h, the cell viability generally increased with increasing concentration as illustrated in Fig. 1b. The highest concentration of 400 mg/l shows a lower viability compared to the control group, while the lower concentrations (50 mg/l, 100 mg/l, and 200 mg/l) demonstrate higher viability. However, at 48 h and 72 h, the viability of cells treated with all concentrations, except for 50 mg/l, decreased compared to that of the control group. The 50 mg/l concentration exhibited a continuous decrease in viability over time.

### Total phenolic content

**Fig. 2:**
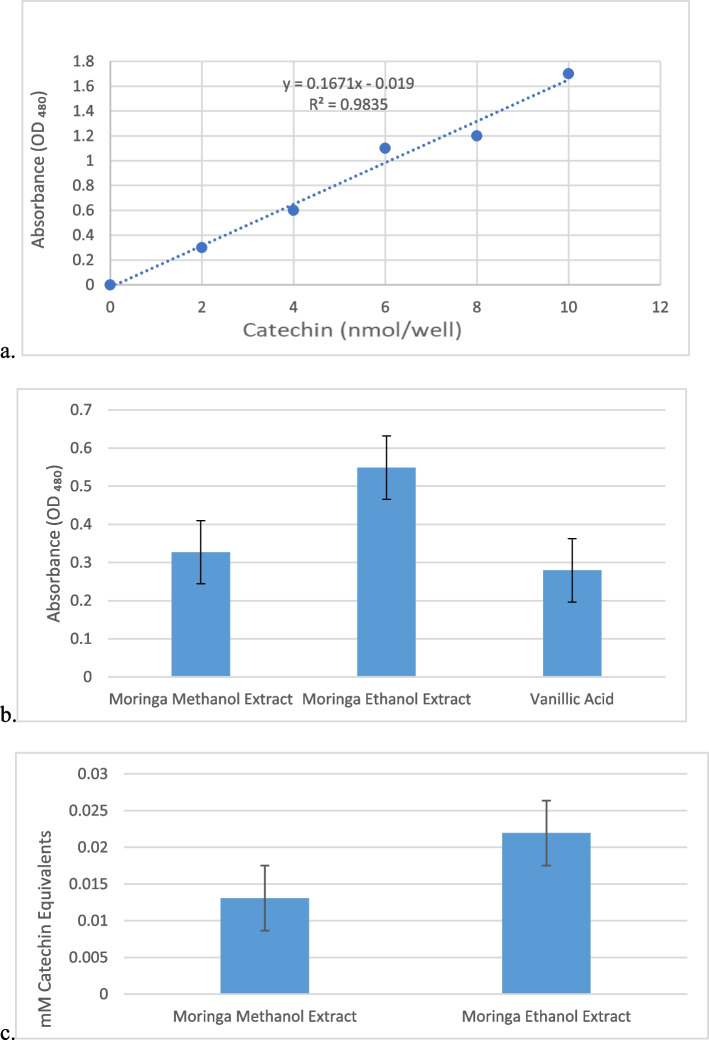
Total Phenolic Content Assay. **a**). Catechin standard curve generated for measuring the total phenol content of the plant extract. **b**). Optical density of *M. oleifera* extract and positive control, vanillic acid. **c**). Catechin equivalent (in mM) of *M. oleifera* and Vanillic Acid (positive control). This represents nmole of phenolic compounds per µl of solution

The optical density of the methanolic extract was found to be 0.3269, while the ethanolic extract exhibited a higher optical density of 0.5487 as shown in Fig. 2b. In comparison, the positive control, vanillic acid had an optical density of 0.2797. These results indicate that both the methanol extract and ethanol extract contain phenolic compounds, as evidenced by their higher optical density values when compared to the control. To determine the concentration of phenolic compounds in the extracts, the optical density values were converted to catechin equivalents using the catechin standard curve in Fig. 2a. Catechin, a well-known phenolic compound, is often used as a reference standard for the quantification of phenolics. The calculated catechin equivalents for the methanol extract and ethanol extract were 0.01307 and 0.02195, respectively as shown in Fig. 2c.

### Antioxidant capacity

**Fig. 3:**
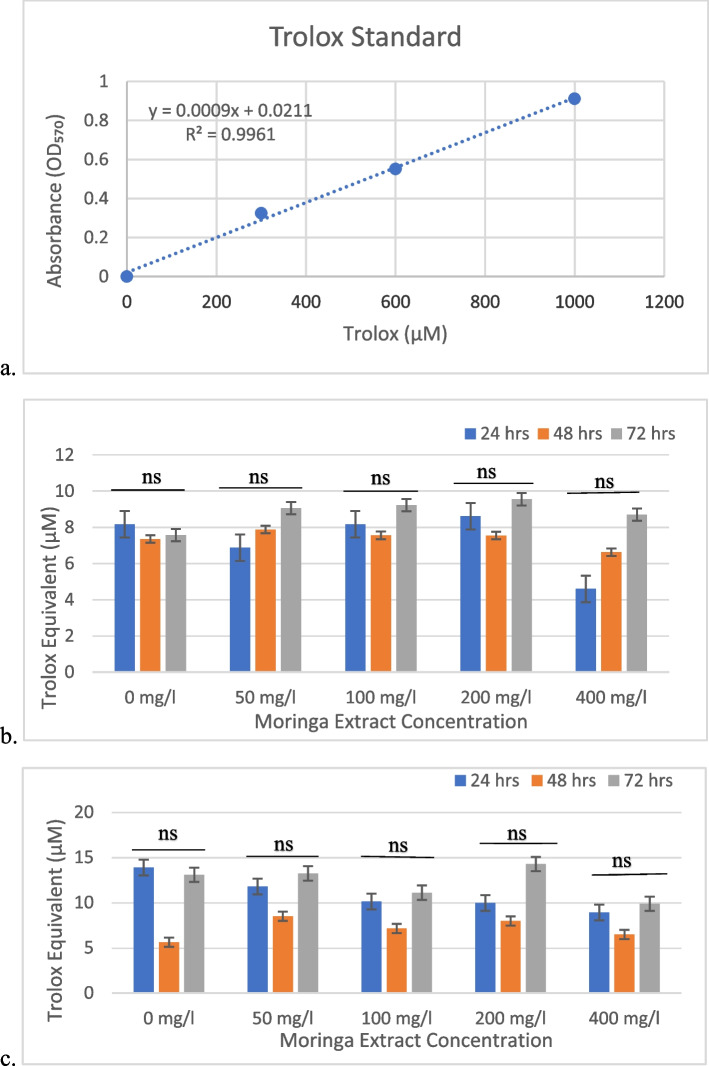
Total antioxidant capacity assay. **a**). Trolox standard curve generated for measuring the TAC of M. oleifera extract. **b**). TAC levels measured after 72 h of treatment with the methanolic extracts. **c**). TAC levels measured after 72 h of treatment with the ethanolic extracts. Values are the mean ± SD *ns: not significant

A trolox standard curve was obtained for this assay (Fig. 3a). The methanol extract of *M. oleifera* exhibits higher antioxidant capacity at various concentrations compared to the control group at 24 h, with the 200 mg/l concentration showing the highest capacity (Fig. 3b). At 48 h, variations in antioxidant capacity were observed across concentrations, and at 72 h, the antioxidant capacity varied further.

For the ethanolic extract, antioxidant capacity varied among concentrations at 24 h, with the control and 50 mg/l concentrations showing the highest capacity (Fig. 3c). At 48 h, variations in antioxidant capacity persisted, and at 72 h, the 200 mg/l concentration displayed the highest capacity.

### Liver enzyme activity

**Fig. 4:**
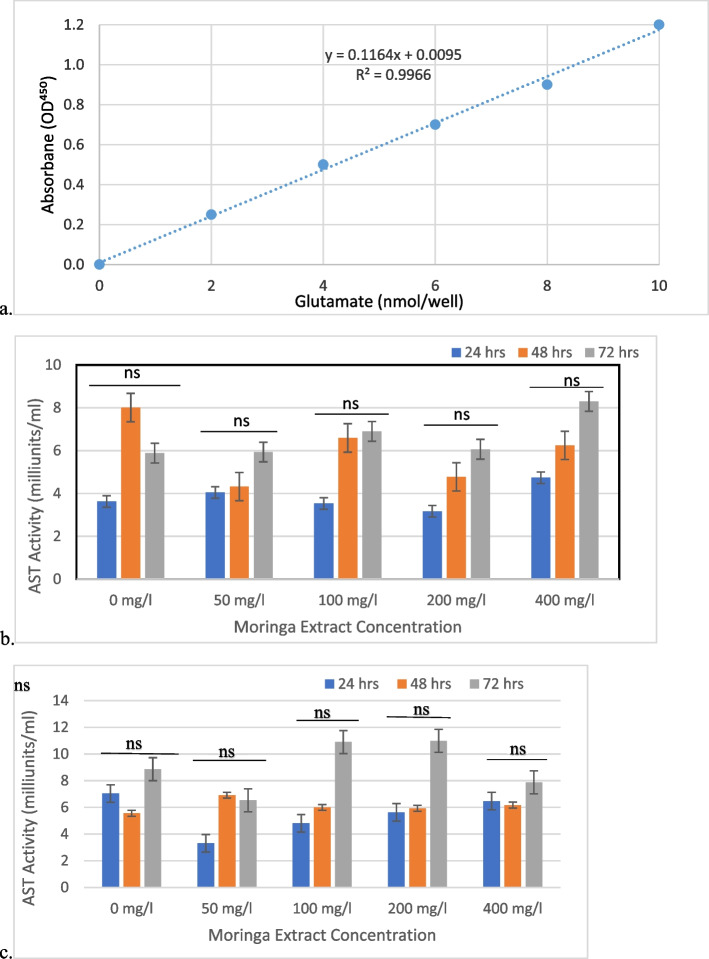
A liver enzyme activity assay was carried out to measure the volume of AST enzymes after treatment with different concentrations of extracts for 72 h. **a**). Glutamate standard curve generated for the measurement of AST levels in treated cells. **b**). AST levels were obtained after treatment with methanolic extract for 72 h. **c**). AST levels were obtained after treatment with ethanolic extract for 72 h. The values are the mean ± SD, *ns: not significant

A glutamate standard curve was obtained for this assay (Fig. 4a). The results obtained from this assay revealed that treatment with *M. oleifera* extracts led to a reduction in liver function markers, specifically AST. Lower levels of this enzyme indicate improved liver cell health and reduced hepatocellular damage. Figure 4b shows that the methanol extract of *M. oleifera* at concentrations of 200 mg/l and 100 mg/l resulted in a reduction in AST levels compared to the control at 24 h. At 48 h, there was a decline in AST levels across all concentrations compared to the control. After 72 h, Fig. 4b shows that AST levels increased across all concentrations compared to the control. ALP levels were also investigated but the results were not remarkable.

For the ethanol extract of *M. oleifera*, at 24 h the AST levels exhibited some variations among the different concentrations (Fig. 4c). The highest AST level was observed at the 200 mg/l concentration, followed by the 400 mg/l concentration (Fig. 4c). The lowest AST level was observed at the 50 mg/l concentration. At the 48-h time point, the AST levels continued to show fluctuations across the concentrations. The 200 mg/l concentration displayed the highest AST level, followed by the 400 mg/l concentration. The AST levels for the 50 mg/l and 100 mg/l concentrations were comparable to each other and slightly higher than those of the control group. At 72 h, the AST levels varied across the concentrations, but the trend was not as clear as that of the previous time points. The 200 mg/l and 400 mg/l concentrations exhibited higher AST levels compared to the control group. ALP levels after treatment with the ethanol extract of *M. ole*ifera were assessed and the results obtained were not remarkable.

## Discussion

In this study, the assessment of the influence of *M. oleifera* extracts on liver cells was facilitated using the HepG2 cell line. This allowed for the evaluation of toxicity through parameters such as cell viability and proliferation [29]. The dose chosen for this study was determined through a thorough examination of the literature in this field [22–24]. Moreover, these studies have encouraged the exploration of higher concentrations to assess their impact on liver cells.

The results obtained from this study demonstrate that the methanolic and ethanol extracts of *M. oleifera* exhibit concentration-dependent and time-dependent effects on cell viability. Lower concentrations of the methanolic extract comparable or slightly higher viability, while the highest concentration had a detrimental effect. Similarly, the ethanol extract initially exhibited increased viability at higher concentrations, but subsequently viability reduction occurred over time, except at the lowest concentration. Consistent with these findings, previous studies have suggested that *M. oleifera* extracts can reduce the viability of HepG2 cells, indicating potential anticancer activity [30, 31].

For the analysis of the total phenolic content in this study, optical density (OD) measurements were obtained for *M. oleifera* methanol extract, *M. oleifera* ethanol extract, and the positive control vanillic acid. The methanol extract yielded an OD of 0.3269, while the ethanol extract displayed a higher optical density of 0.5487. By comparison, the positive control vanillic acid recorded an OD of 0.2797. These results corroborate the presence of phenolic compounds in both extracts, as indicated by their elevated OD values compared to the control. Conversion of OD values to catechin equivalents, a widely recognized phenolic compound standard, revealed catechin equivalents of 0.01307 for *M. oleifera* methanol extract and 0.02195 for *M. oleifera* ethanol extract.. The presence of phenolic compounds in these extracts is of paramount interest due to their potential health benefits, including antioxidant and anti-inflammatory properties associated with reduced risk of chronic diseases [32].

The results obtained from the antioxidant activity assay demonstrate that both methanol and ethanolic extracts of *M. oleifera* exhibit antioxidant activity across various concentrations and time points. The methanol extract exhibited a higher antioxidant capacity than the ethanolic extract (Fig. 3b and c). While the methanol extract maintained consistent antioxidant capacity, especially at higher concentrations, the ethanolic extract displayed more intricate patterns. This suggests that the choice of solvent for extraction significantly influences the antioxidant potential of the extract. These observations align with prior research indicating the antioxidant capacity of *M. oleifera* extract [33–36].

The liver enzyme activity assay results indicate the ability of *M. oleifera* extracts to mitigate liver function markers, notably AST and ALP enzymes. The methanol extract, demonstrated decreased AST levels at concentrations of 200 mg/l and 100 mg/l after 24 h suggesting potential liver cell protection. However, at 48 h, AST levels decreased across all concentrations, suggesting temporary suppression of liver function, followed by an increase after 72 h, indicating a regenerative response. For ALP levels, reductions were observed at the 200 mg/l concentration after 24 h, and at 200 mg/l and 50 mg/l after 48 h and 72 h. The reduction in ALP levels further suggests that the *M. oleifera* extract, particularly at 200 mg/l, may contribute to improved liver function by minimizing hepatocellular damage.

The ethanol extract exhibits varying effects on AST levels at different concentrations and time points. At 24 h, the 200 mg/l concentration showed the highest AST level, followed by 400 mg/l, while the lowest level was observed at 50 mg/l. At 48 h, AST levels continued to fluctuate, with the 200 mg/l concentration having the highest level. At 72 h, the relationship between AST levels and concentrations became less distinct. Regarding ALP levels, at 24 h, the 50 mg/l and 200 mg/l concentrations of the *M. oleifera* extract reduced ALP levels. At 48 h, the 50 mg/l and 400 mg/l concentrations decreased ALP levels, while the 100 mg/l and 200 mg/l concentrations showed minor changes. After 72 h, most concentrations exhibited a slight decrease in ALP levels compared to the control group, except for the 200 mg/l concentration.

These results underscore the potential of both methanol and ethanol extracts of *M. oleifera* in enhancing liver cell health and mitigate hepatocellular damage, depending on concentration and treatment duration. The implications of these findings are significant for liver health and warrant further investigation.In summary, this study investigated the effects of *M. oleifera* extracts on liver cells, particularly in an in vitro setting using a cancer cell line. The outcomes revealed concentration-dependent and time-dependent impacts on cell viability. We observed that lower extract concentrations resulted in similar or slightly improved cell viability, while the highest concentration had a negative effect. Both extracts contained phenolic compounds, with the ethanol extract displaying a higher concentration. In terms of antioxidant activity, the methanol extract exhibited greater potential. Additionally, the extracts were found to lower the levels of certain liver function markers, suggesting potential cellular protection.

## Conclusion

In conclusion, the insights obtained during this study revealed the intricate interplay between the characteristics of the extracts and the extraction methods. Our findings have contributed to the current body of knowledge on *M. oleifera*. This has also extended our understanding of how *M. oleifera* extracts could contribute to liver health. However, it is crucial to note that our study exclusively focused on cancer cells in a controlled environment, which means we cannot jump to conclusions about their hepatoprotective properties. While this study sets the stage for future explorations of the complex interactions between *M. oleifera* extracts and liver cells, further comprehensive research is needed before we can definitively establish their potential for enhancing liver health.

## Limitations

The reliance on the HepG2 cell line, although a commonly used model, might not fully mirror real physiological responses. Additionally, the controlled in vitro environment fails to encompass the complexities of the human body. The study's short duration of exposure and focus on a single cancer cell line, HepG2, could limit the generalizability of findings to different cell types or long-term scenarios.

## Data Availability

The datasets generated and/or analysed during the current study are available from the corresponding author upon reasonable request.
